# Integrated self-management support provided by primary care nurses to persons with chronic diseases and common mental disorders: a scoping review

**DOI:** 10.1186/s12912-022-01000-2

**Published:** 2022-08-02

**Authors:** Jérémie Beaudin, Maud-Christine Chouinard, Ariane Girard, Janie Houle, Édith Ellefsen, Catherine Hudon

**Affiliations:** 1 Faculté de Médecine Et Des Sciences de La Santé, Université de Sherbrooke, 12e Avenue Nord, Sherbrooke, Québec 3001J1H 5N4 Canada; 2 Faculté Des Sciences Infirmières, Université de Montréal, Pavillon Marguerite-d’Youville, C.P. 6128 succ. Centre-ville, Montréal, Québec H3C 3J7 Canada; 3 Faculté de Médecine, Université Laval, VITAM Research Center On Sustainable Health, 2601, Chemin de La Canardière (G-2300), Québec, Québec G1J 2G3 Canada; 4 Département de Psychologie, Université du Québec À Montréal, case postale 8888, succ. Centre-ville, Montréal, Québec H3C 3P8 Canada; 5 École des sciences infirmières, Faculté de Médecine Et Des Sciences de La Santé, Université de Sherbrooke, 12e Avenue Nord, Sherbrooke, Québec 3001J1H 5N4 Canada

**Keywords:** Self-management support, Nurses, Primary care, Integrated care, Common mental disorders, Chronic diseases, Scoping review, Clinical integration

## Abstract

**Aim:**

To map integrated and non-integrated self-management support interventions provided by primary care nurses to persons with chronic diseases and common mental disorders and describe their characteristics.

**Design:**

A scoping review.

**Data sources:**

In April 2020, we conducted searches in several databases (Academic Research Complete, AMED, CINAHL, ERIC, MEDLINE, PsycINFO, Scopus, Emcare, HealthSTAR, Proquest Central) using self-management support, nurse, primary care and their related terms. Of the resulting 4241 articles, 30 were included into the analysis.

**Review methods:**

We used the Rainbow Model of Integrated Care to identify integrated self-management interventions and to analyze the data and the PRISMS taxonomy for the description of interventions. Study selection and data synthesis were performed by the team. Self-management support interventions were considered integrated if they were consistent with the Rainbow model’s definition of clinical integration and person-focused care.

**Results:**

The 30 selected articles related to 10 self-management support interventions. Among these, five interventions were considered integrated. The delivery of the interventions showed variability. Strategies used were education, problem-solving therapies, action planning, and goal setting. Integrated self-management support intervention characteristics were nurse-person relationship, engagement, and biopsychosocial approach. A framework for integrated self-management was proposed. The main characteristics of the non-integrated self-management support were disease-specific approach, protocol-driven, and lack of adaptability.

**Conclusion:**

Our review synthesizes integrated and non-integrated self-management support interventions and their characteristics. We propose recommendations to improve its clinical integration. However, further theoretical clarification and qualitative research are needed.

**Implication for nursing:**

Self-management support is an important activity for primary care nurses and persons with chronic diseases and common mental disorders, who are increasingly present in primary care, and require an integrated approach.

**Impact:**

This review addresses the paucity of details surrounding integrated self-management support for persons with chronic diseases and common mental disorders and provides a framework to better describe its characteristics. The findings could be used to design future research and improve the clinical integration of this activity by nurses.

## Background

Globally, physical chronic diseases (CD) are responsible for approximately 70% of deaths and their prevalence is increasing [1]. Also recognized as chronic health conditions [2], common mental disorders (CMD), i.e., anxiety and depressive disorders, are also highly prevalent worldwide [3]. The concomitance of CD and CMD causes several negative effects such as deterioration of the affected individuals’ overall health [4], increased morbidity [5], increased mortality [6], as well as a significant burden on the health care system involving increased service utilization and the potential for health care fragmentation [7]. To overcome these effects, the person has an essential role to play in the day-to-day management of his/her CD and CMD (self-management) and health care professionals can play an important role in supporting the individual through self-management support (SMS) [8]. A study of the needs of people with CD and CMD revealed complex self-management and issues of limited access to mental health care, long wait times, fragmentation of care and services, and an increased burden on the individual with respect to his/her care [9]. As an answer to these problems, integrated SMS has the potential to improve self-management for both CD and CMD [10]; to better meet the complex needs of these individuals; to decrease fragmentation of care and it is consistent with current priorities for improving primary care for this clientele [11]. However, current guidelines for SMS of persons with CD and CMD (e.g., NICE guideline for persons with depression and chronic conditions) do not specify the components of integrated self-management support and how to improve clinical integration of this important primary care nurse activity [11, 12]. Therefore, this review was conducted to shed light on this matter.

According to the Institute of Medicine [13], SMS can be defined as “*the systematic provision of education and supportive interventions by health care staff to increase patients’ skills and confidence in managing their health problems, including regular assessment of progress and problems, goal setting, and problem-solving support”*. SMS encompasses several key components, such as an individualized educational care plan, including self-management skills development with various strategies, personalized educational materials, feedback, and social support [14]. Benefits are linked to SMS of CD such as improvement in health-related quality of life, cholesterol, blood pressure [15] and CMD such as improvement in self-efficacy, reduction in depressive and anxiety symptomatology, and the number of relapses [16]. The scientific literature suggests and encourages self-management and SMS of CD and CMD, especially by nurses [17].

It is widely recognized that primary care is a gateway to chronic health condition prevention, treatment and follow-up for the majority of people [18]. Primary care nurses play an essential role in the care management of people with CD and CMD, and they perform several important activities: global health assessment of the person, health promotion (including health education, self-management support, screening and prevention), collaboration with team members and care coordination [19]. Among these, SMS is one of the main activities practiced by nurses in primary care for CD [19]. Moreover, along with general practitioners, nurses are involved the most in SMS of CD and CMD compared to other professionals and they represent a growing workforce in primary care [17, 20, 21]. Many reviews provide positive evidence of nurse-led SMS of persons with chronic conditions, supporting its use in primary care [15, 22]. However, although SMS should be person-centered, it is more often practiced in the context of specific diseases [20], in silos (CD or CMD), especially to the benefit of physical CD [23], and with a lack of coordination [10].

The concept of integration is proposed as a way to overcome the problem of fragmentation of care. Integration is an approach that aims to improve quality of care through the coherent coordination of different person-centered health care and services [24]. From the person’s perspective, integration of care can be summarized as: “My care is planned with people who work together to understand me and my carer(s), put me in control, coordinate and deliver services to achieve my best outcomes.” [25]. In the field of integrated care, many models and taxonomies were developed to serve various purposes with different scopes [25, 26]. Among these, the Rainbow Model of Integrated Care (RMIC) [27] often stands out [24, 28] because it offers both a conceptual model and a taxonomy [29] of what constitutes integrated care and it was developed specifically for primary care settings [27]. In addition, this model clearly defines integration at the clinical level (i.e., clinical integration) [27]. Its conceptual foundation (i.e., domains, types of integration, processes) is also shared by other models of integrated care [26, 30, 31].

**Fig. 1:**
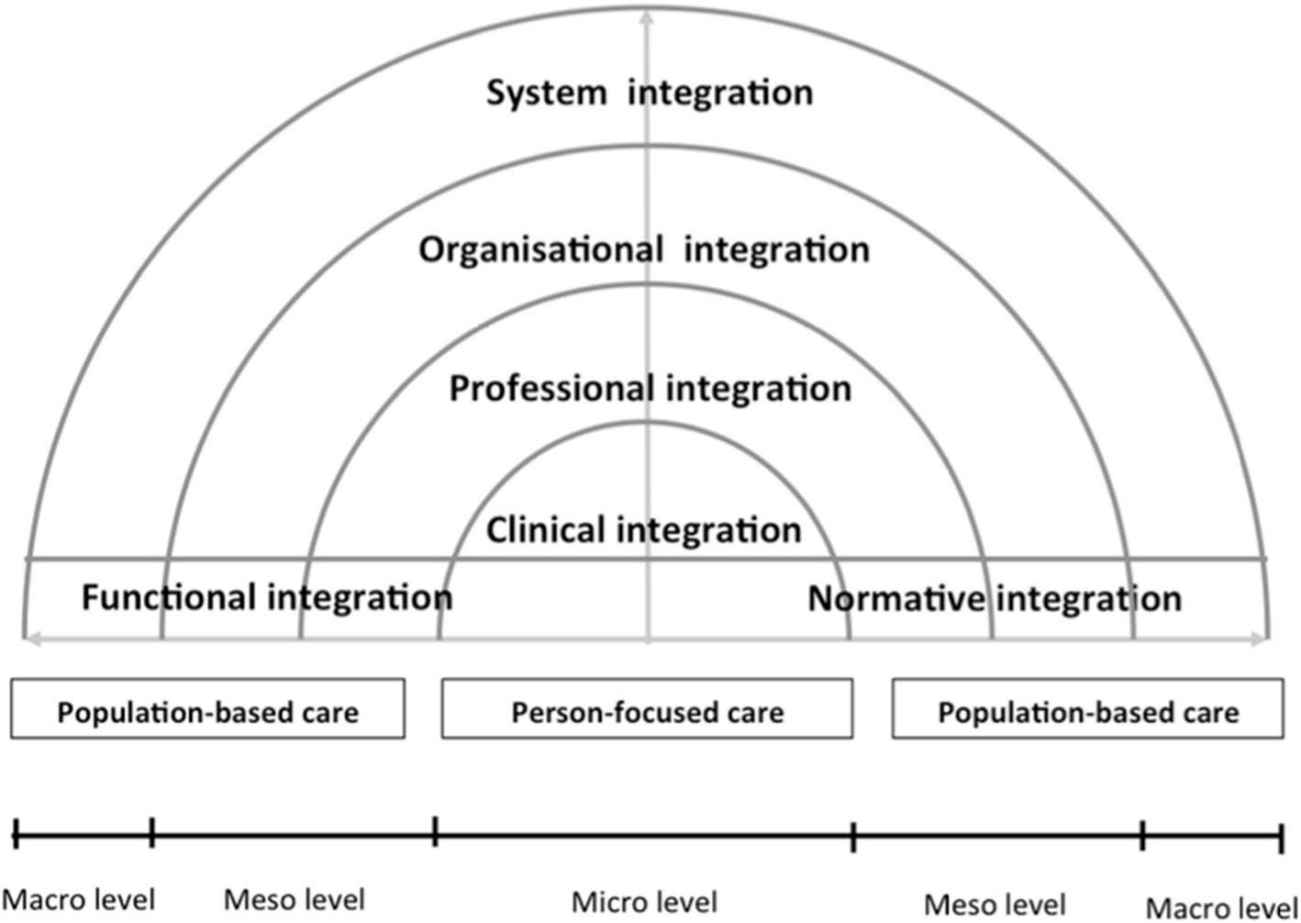
Rainbow Model of Integrated Care (original from Valentijn et al. [27])

Valentijn et al.’s RMCI [27] defines integration of care within different integration processes (see Fig. 1). First, the model defines two scopes of integration: population-based (integration of health services to meet the needs of a population) and individual (integration of care to meet the biopsychosocial needs of an individual). Next, the model distinguishes between the domains of integration processes to provide a comprehensive continuum of care for individuals and populations. These domains are: systemic (policy arrangements); organizational (inter-organizational partnerships); professional (inter-professional partnerships); functional (support mechanisms and communication tools); normative (cultural frame of reference mutually respected by all); and clinical. Clinical integration is defined as “Coordination of person-focused care for a complex need at stake in a single process across time, place and discipline.” [29].

For this review, in line with Valentijn’s definition, integrated SMS involves clinical integration of care by a nurse or other health professional with a person-focused care approach [27]. Valentijn et al. define person-focused care as a “perspective to improve someone’s overall well-being and not focus solely on a particular condition.” [27] (p. 7). To be person-focused, SMS interventions must present a biopsychosocial perspective of health and be based on the person’s preferences, needs, and values. In addition to being person-focused, clinically integrated SMS interventions must be based on co-creation of the care process between the nurse and the person, have shared responsibility by demonstrating joint agreement on clinical management, and have the person coordinate his/her own care when possible. However, a clear description of what constitutes integrated SMS, including its characteristics, is still lacking.

The aim of this scoping review was to map integrated and non-integrated self-management support interventions provided by primary care nurses to persons with CD and CMD and their characteristics. Research questions were as follows:

1. What are the integrated and non-integrated SMS interventions, according to Valentijn’s model, for persons with concurrent CD and CMD performed by primary care nurses?
2. What are the characteristics of integrated and non-integrated SMS interventions?

## Methods

### Design

Arksey and O’Malley’s [32] scoping review method, enhanced by Levac et al. [33], was used as it is a preferred method for mapping the literature of a complex domain of interest and for examining and summarizing findings. This scoping review was conducted in five steps, as described below. The PRISMA-ScR Checklist was used to guide the writing of this article [34].

### Identifying relevant studies

The concepts of self-management support, nurse and primary care and their related terms were used to develop the search strategy in collaboration with a librarian (see Table 1). The following databases were searched up to April 2020 without time limits: Academic Research Complete, AMED, CINAHL, ERIC, MEDLINE, PsycINFO, Scopus, Emcare, HealthSTAR, Proquest Central. We used a variety of related terms in our search strategy to compile as many articles as possible about SMS. To be included, studies had to: 1) present a primary care nurse SMS intervention targeting both physical and common mental health conditions; 2) include a qualitative, quantitative, mixed methods or research protocol design; and 3) be in English or French. Articles were excluded if they: 1) included a SMS intervention targeting only physical or only mental conditions; or 2) included a specific client population (i.e., severely mentally ill, pediatric, obstetric, HIV/AIDS, home care, oncology, or palliative care). Although certain specific clientele may involve concurrent CD and CMD, they tend to need more specialized care than primary care and are not targeted by the scope of this review.

**Table 1:** Literature search strategy

Key concepts	Research strategy
**Support for self-management**	TI-AB-SU ((“self-management” OR “self management” OR “self care” OR “self-care” OR “self-help” OR “self help”) N2 (support or education)) OR “collaborative care” OR ((MM “self-management”) OR (MM “self care”) OR (MH “models, nursing”) OR (MH “self concept”) OR (MH “self-assessment”) OR (MH “self-examination”) OR (MH “self administration”) OR (MH “self-control”) OR (MH “self efficacy”)))
AND	
**Nurse**	TI-AB-SU ((nurs*) OR ((MM “nursing”) OR (MM “nursing care”) OR (MM “nurses”) OR (MM “nurses, community health”) OR (MM “family nurse practitioners”) OR (MM “nurse practitioners”) OR (MM “nurse specialists”) OR (MM “nurse clinicians”)))
AND	
**Primary care**	TI-AB-SU (((primary N2 care) OR “community care” OR “community health service*” OR “ambulatory care”)) OR (MM “primary health care”) OR (MM “primary care nursing”) OR (MM “primary nursing”))

### Study selection

**Fig. 2:**
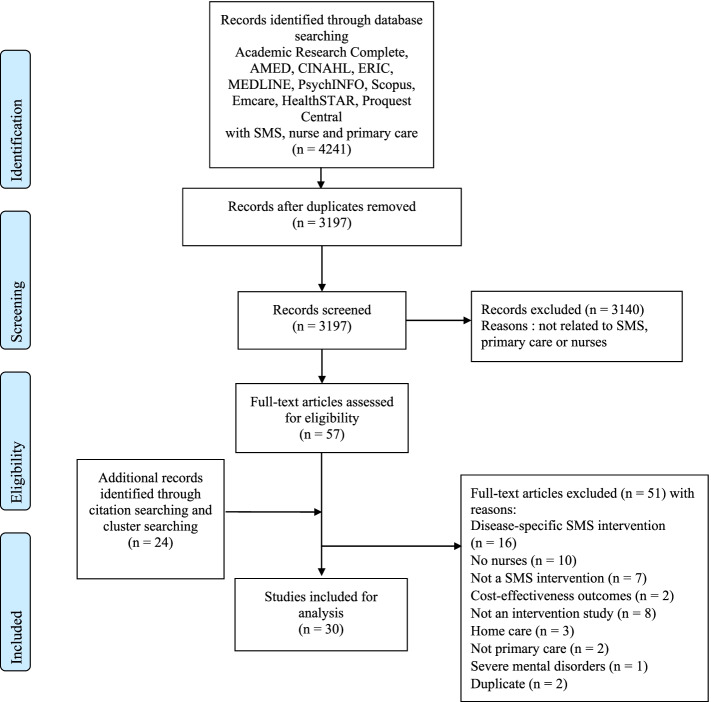
Flowchart. Adaptation of Moher D, Liberati A, Tetzlaff J, Altman DG, The PRISMA Group (2009). Preferred Reporting Items for Systematic Reviews and Meta-Analyses: The PRISMA Statement. PLoS Med 6(7): e1000097. https://doi.org/10.1371/journal.pmed1000097

The literature search identified 4241 articles. The Rayyan online platform was used for the team sorting process and Endnote X9 software was used for reference management. After removing any duplicates, a first sort was performed by the first author (*n* = 3197 articles) and 57 articles were read by two authors. Citation and cluster searches identified 24 additional articles to be read, bringing the total to 81 articles read in full. In the end, 30 articles were included for analysis (see Fig. 2).

### Charting the data and collating, summarizing, and reporting the results

Descriptive characteristics of the included studies were extracted including the aim of the study; design; setting; theoretical foundations of SMS; mode of delivery, frequency, and duration of SMS; targeted population; and SMS strategies. Descriptive characteristics related to SMS were extracted and summarized using the PRISMS taxonomy of self-management support [35]. This taxonomy conceptualizes SMS into 14 potential components and four overarching dimensions (mode of delivery; personnel delivering the support; targeted population; and intensity, frequency and duration of the intervention) [35]. This taxonomy was chosen because its broad conceptualization of SMS makes it easy to use and to report on SMS interventions and it was developed and tested using a rigorous and transparent process for many chronic conditions, including physical CD and CMD [35].

For the first research question, which sought to map integrated and non-integrated SMS interventions, articles were analyzed deductively using the categories identified by the clinical integration and person-focused care definitions of the Valentijn et al. model [27]. Predetermined charting forms, that can be based on a theoretical model, were often used in previous scoping reviews [36]. Using this model, we were able to clearly define the essential elements that are needed to consider SMS interventions, whether they are integrated or not. SMS interventions were considered integrated if they fit every category of clinical integration defined by Valentijn’s model [27], shown in Table 2.

**Table 2:** Definitions of person-focused care and clinical integration based on Valentijn’s Rainbow Model of Integrated Care [27]

	**Categories**		**Definitions**
**Clinical integration**	**Person-focused care**	**Biopsychosocial perspective**	“The first feature, person-focused care, reflects a biopsychosocial perspective of health, as it acknowledges that health problems are not synonymous to biological terms, diagnoses or diseases […] It bridges the gap between medical and social problems as it acknowledges that diseases are simultaneously a medical, psychological and social problem.” (p. 4)
		**Care based on personal preferences, needs and values**	“Moreover, person-focused care is based on personal preferences, needs, and values (i.e., understanding the personal meaning of an illness).” (p. 4) “Professionals have to take proper account of the needs of individuals, so that services provided are matched to their needs. […] Emphasis should be placed on a person’s needs.” (p. 7)
	**Co-creation of care process**		“This also encloses the important aspect of the patient as a co-creator in the care process” (p. 7)
	**Shared responsibility and common agreement**		“[…] with shared responsibility between the professional and the person to find a common ground on clinical management” (p. 7)
	**Person coordinating his/her own care**		“Emphasis should be placed on a person’s needs, with people coordinating their own care whenever possible” (p. 7)

For the second research question on the characteristics of integrated and non-integrated SMS interventions, an iterative deductive-inductive qualitative thematic analysis was conducted, as recommended by Levac et al. [33, 37]. On the one hand, deductively following the categories of person-focused care and clinical integration defined by the Valentijn et al. model [27], and on the other hand, inductively coding emerging themes for each category [37]. Data extraction and analysis were performed using MAXQDA2020 software.

## Results

### Descriptive characteristics of the included studies

Ranging from 2004 to 2017, the 30 articles referred to ten intervention studies. An average of 3 articles were published per intervention study. Each article (*n* = 30) was thoroughly analyzed and was linked to its intervention. Table 3 provides a detailed description of the intervention studies (*n* = 10) instead of describing each article individually. Eight of the 10 studies were published in the last decade. The studies were conducted in the United States [38–51], the United Kingdom [52–60], Australia [61, 62], the Netherlands [63, 64] and Canada [65–67]. Most studies (*n* = 8) were randomized controlled trials, while one was a quasi-experimental before-and-after study and only one was specifically a qualitative study. Most of the studies were conducted in 4 to 27 primary care clinics, though one America-wide study involved 172 clinics.

**Table 3:** Description of included studies

Study name Country	Aim of the study	Design	Setting	SMS theoretical foundations	SMS mode of delivery	SMS frequency and duration	Targeted population	SMS strategies
**SCAMP study** [48–51] **USA**	To determine if a combined pharmacological and behavioral intervention improves both depression and pain in primary care patients with musculoskeletal pain and comorbid depression	Protocol [50] RCT [51] Qualitative study [48] longitudinal analysis [49]	11 veteran affairs and university primary care clinics	Stepped-care protocol based on: Stanford SM program, Social Cognitive Theory, SCAMP conceptual model	Face-to-face and by phone	12 weeks antidepressants (step 1), 6 × 30 min Pain SM sessions over 12 weeks, 2 additional contacts occurring at 8–10 months (medication and pain self-management adherence)	Primary care patients with comorbid musculoskeletal pain and depression (*n* = 250) Adult patients with musculoskeletal pain in the lower back, hip or knee and comorbid clinical depression The depression had to be of at least moderate severity, that is, a PHQ-9 score ≥ 10 and endorsement of depressed mood and/or anhedonia. Depression severity was assessed using SCL-20. Anxiety was assessed with GAD-7	• Education on pain SM • Pain SM manual • Problem-solving therapy • Goal setting • Action-planning • Condition monitoring • Feedback • Behavior monitoring • Relaxation • Deep breathing • Positive thinking • Evaluating non-traditional treatments • Practical support to SM • Health behavior advice
**COMPASS study** [41–43] **USA**	To disseminate and implement an evidence-based collaborative care management model for patients with both depression and poorly controlled diabetes and/or cardiovascular disease across multiple, real-world diverse clinical practice sites	Before-after experimental study [43] Quantitative descriptive [41] Intervention development and implementation [42]	Multistates medical groups (18 care systems, 172 primary care clinics) Integrated systems	Chronic Care Model (collaborative care) and TEAMcare as base model	Face-to-face and by phone	Duration: 3–12 months Intensity: at least 1x/month Active management phase: weekly (1st month) and then frequency gradually extended to monthly to every 3 months	Active depression (PHQ-9 of at least 10) and 1 poorly controlled medical condition (diabetes or high blood pressure)	• Education • Problem solving • Goal setting • Behavioral activation • Support for treatment adherence • Motivational interviewing • Brief intervention for misuse of alcohol or other substances • Social support • Systematic case review • Condition monitoring
**UPBEAT-UK study** [52–57] **UK**	To explore the relationship between CHD and depression in a GP population and to develop nurse-led personalised care (PC) for patients with CHD and depression	Literature review [53] Intervention development [52] Qualitative descriptive [55] Pilot RCT [56] Pilot RCT protocol [54] UPBEAT-UK research program [57]	17 general practices in South London	Practice nurse-delivered personalized care intervention Own SMS definition: “Enabling patients to take better care of themselves” [56]	Face-to-face and by phone	Weekly, 15 + min sessions Duration: 6 months. Frequency: depending on needs	Adults with symptomatic CHD (registered on GP CHD QOF register and reporting chest pain), reporting depression symptoms were eligible. HADS-20 (8 or more for depression), modified Rose Angina Questionnaire for CHD	• Education (provide information) • Problem solving • Goal setting • Action planning • Social support • Case review • Self-monitoring • Motivational interviewing • Cognitive behavioral therapy
**Pathways study** [44–47] **USA**	1) To investigate prevalence and impact of depression in patients with diabetes enrolled in a health maintenance organization using a population-based investigation; and 2) To test the effectiveness of collaborative care interventions in improving the quality of care and outcomes of depression among patients with diabetes in primary care within a randomized controlled trial	Protocol [46] RCT [45] Qualitative descriptive [44] Secondary analysis [47]	9 primary care clinics in Western Washington	Collaborative Care Model based on the IMPACT study	Face-to-face and by phone	Step 1: 0–12 weeks, follow-up twice a month, 30-60 min Step 2: 12–24 weeks, once or twice/month depending on good/bad outcomes, 30 min Step 3: 24–52 weeks, once or twice/month, depending on good/bad outcomes, 30 min	Adults with diabetes and depression (PHQ greater than or equal to 10, SCL-20 depression mean item of 1.1 or greater) or dysthymia	• Patient education and support • Problem-solving • Goal setting • Action planning • Behavioral activation • Monitoring of adherence and outcomes • Medication management support • Motivational approach • Counselling • Case review
**TEAMcare study** [38–40] **USA**	To determine whether a primary care based, care management intervention for multiple conditions would improve medical outcomes and depression scores among patients with major depression and poorly controlled diabetes, coronary heart disease, or both	RCT and results [39] RCT results [38] RCT results [40]	14 primary care clinics in Group Health Cooperative in Washington state	Elements from: collaborative care, the Chronic Care Model and treat-to-target strategies (timely pharmacotherapy adjustment to achieve treatment goals) SMS is defined self-care support [38]	Face-to-face and by phone	Structured visits every 2–3 weeks until targets reached, every 4 weeks afterward (maintenance)	Adults with diagnoses of diabetes, coronary heart disease, or both, and depression (PHQ-2 3 or greater; PHQ-9 10 or greater)	• Provision of self-care materials (self-help book, booklet, a video compact disk) • Problem solving treatment for primary care (PST-PC) • Goal setting • Behavioral activation • Medication adherence strategies • Condition monitoring • Motivational coaching • Support for self-care • Support for self-monitoring • Moral boosting • Case review • SMS materials
**TEAMcare-PCN** [65–67] **Canada**	To evaluate the comparative effectiveness of a collaborative model of care for patients with type 2 diabetes and depressive symptoms in the Canadian primary care setting while also determining the value of screening for depression itself when compared with usual care delivered outside the trial setting	Protocol [65] Controlled pragmatic trial [66] Qualitative implementation evaluation [67]	4 primary care networks in Alberta	Adaption of Collaborative Care Model from TEAMcare approach	Face-to-face and by phone	Follow-up 1-2x/month, over 12-month period	Adults with type 2 diabetes and under the care of a primary care network family physician, Score > = 10 on the PHQ-9, speak English and have adequate hearing to complete telephone interviews and be willing and able to provide written informed consent to participate	• Patient education • Problem-solving therapy • Action planning • Shared care plan • Behavioral activation • Treatment adherence monitoring • Motivational interviewing
**CAREplus study** [59, 60] **UK**	To evaluate a whole-system primary care-based complex intervention, called CARE Plus, to improve quality of life in multimorbid patients living in areas of very high deprivation	Protocol and pilot testing [59] RCT [60]	8 general practices in Glasgow	The CARE plus approach (holistic patient-centred care approach) and SMS	Face-to-face	30–45 min consultations	Adults with multimorbidity (average of 5 CD) (including CD and CMD) Depression/anxiety were present for nearly 70% of participants	• Education with SMS materials (mindfulness-based stress management CDs, CBT-derived self-help booklet, written material) • Goal setting • Action planning • Motivational interviewing
**Trueblue study** [61, 62] **Australia**	To determine the effectiveness of collaborative care in reducing depression in primary care patients with diabetes or heart disease using practice nurses as case managers	RCT protocol [61] RCT [62]	11 Australian general practices	Adaptation of IMPACT Collaborative Care Model, including stepped-care (psychotherapy or pharmacotherapy)	Face-to-face	45 min session every 3 months for 1 year	Adults with comorbid depression (PHQ-9 5 or greater) and heart diseases/diabetes	• Education and educational SMS materials • Problem-solving • Goal setting • Action planning • Behavioral techniques • Health behavior advice
**Step-dep study** [63, 64] **The Netherlands**	To investigate whether a pragmatic nurse-led stepped-care program is effective in reducing the incidence of major depressive disorders at 12-months follow-up in comparison to usual care among patients with type 2 diabetes and/or coronary heart disease and subthreshold depression (Step-Dep trial)	Cluster RCT protocol [64] Pragmatic cluster RCT [63]	27 primary care centers	Stepped-care intervention based on van’t Veer-Tazelaar Model	Face-to-face and by phone	4 steps of 3 months each	Adults with subthreshold depression (PHQ-9 six or greater) and NOT major depression according to DSM-IV measured with MINI and diabetes and/or heart diseases	• Provide information (step 1) • Guided self-help course (step 2) • Problem-solving treatment (max. 7 sessions during 12 weeks, step 3) • Motivational interviewing • Condition monitoring
**Langer study** [58] **UK**	To outline the intervention; to use the accounts of patients who experienced the intervention to characterize its main features; to use the accounts of primary care staff to understand how the intervention was incorporated into primary care; and to reflect on implications for meeting psychosocial needs of patients with COPD in UK general practice	Qualitative study [58]	6 primary care practices	Collaborative care, Whole System Framework and cognitive-behavioural approaches Liaison health workers (LHW) are nurses added to the primary care clinics	Face-to-face, at-home or by phone	Not specified	Adults with COPD and common mental disorders and psychosocial problems (QOF diagnosis with at least 1 QOF diagnosis of depression, social isolation, and chronic or recent psychosocial stressors)	• Education and information (medication management, SMS materials) • Problem-solving • Goal setting • Psychosocial interventions • Cognitive behavioral therapy • Health behaviour advice • Social support • Relaxation techniques • Practical support

*CHD* Chronic heart disease, *COPD* Chronic obstructive pulmonary disease, *QOF* Quality and Outcomes Framework, *RCT* Randomized controlled trial, *SM* Self-management, *SMS* Self-management support

### Participants targeted by SMS

The participants targeted by the majority of interventions were people with multimorbidity, involving various physical CD (chronic musculoskeletal pain, diabetes, high blood pressure, coronary heart disease, COPD) and common mental disorders (subthreshold depression, major depression, dysthymia, anxiety) [38–58, 61–67]. One intervention [59, 60] involved multimorbid patients (average of 5 CD) with a majority of patients suffering from depression and/or anxiety, and another [58] had additional inclusion criteria related to social isolation and recent or chronic psychosocial stressors.

### Theoretical foundations of the studies

As theoretical bases for their interventions, a majority of studies used the Collaborative Care Model [38–47, 58, 61, 62, 65–67]. A few studies used various forms of stepped-care protocol [44–51, 61–64], Social Cognitive Theory [48–51], SCAMP conceptual model [48–51], Personalized Care Intervention [52–57], Treat-to-target strategies [65–67], CARE approach [59, 60] and Whole System Frameworks [58]. As for the specific concept of SMS, only two studies defined the concept of SMS, either by referring to Lorig and Holman’s definition of self-management (SM) [8, 57] or by defining it as self-care support [39].

### Mode of delivery, frequency, duration and strategies of SMS

SMS was administered either in person or by telephone. The duration of interventions ranged from 3 to 12 months, with variable frequencies depending on either the person’s needs and/or predetermined frequencies ranging from 4 to 24 sessions per year. Sessions lasted 15 to 45 min. Several techniques were used during the SMS interventions. Table 3 shows a summary of the strategies used by each SMS intervention. SMS interventions included many components of the PRISMS taxonomy of SMS [35]. Table 4 presents a summary of the strategies used and their related components.

**Table 4:** PRISMS taxonomy components and self-management support strategies [35]

Components	SMS strategies
A1. Information about condition and/or its management	**Therapeutic education** [39, 42, 45, 51, 57–59, 62, 63, 66]
A3. Provision of/agreement on specific clinical action plans and/or rescue medication	**Actions plans** [39, 42, 45, 51, 57–59, 62, 63, 66]
A4. Regular clinical review	**Evaluating non-traditional treatments** [51] **Case review** [39, 42, 45, 57]
A5. Monitoring of condition with feedback	**Monitoring of adherence (behavior and/or medication)** [39, 45, 51, 66] **Monitoring of condition with feedback** [39, 42, 45, 51, 63] **Support for self-monitoring** [39, 57]
A6. Practical support with adherence – medication or behavioral	**Medication management support** [39, 42, 45]
A7. Provision of equipment	**SMS educational materials** [39, 51, 58, 59, 62, 63]
A10. Training/rehearsal for everyday activities	**Support for self-care** [39]
A11. Training/rehearsal for practical self-management activities	**Practical support of self-management** [39, 51]
A12. Training/rehearsal for psychological strategies	**Problem-solving therapy** [39, 42, 45, 51, 57, 58, 62, 63, 66] **Goal setting** [39, 42, 45, 51, 57–59, 62, 63, 66] **Action planning** [39, 42, 45, 51, 57–59, 62, 63, 66] **Relaxation techniques** [51, 58] **Talking therapies/counselling** [51, 57] **Informal counselling** [57] **Positive thinking** [51] **Emotional management** [51] **Motivational interviewing** [39, 42, 45, 57, 59, 63, 66] **Negotiation methods** [39] **Behavioral activation and techniques** [39, 42, 45, 62, 66] **Morale-boosting strategies** [39] **Mindfulness-based approaches** [59] **Cognitive behavioral therapy (cognitive restructuring, 10 min CBT, mini-CBT)** [57, 58]
A13. Social support	**Psychosocial interventions and social support** [42, 57, 58]
A14. Lifestyle advice and support	**Brief interventions for misuse of alcohol or other substances** [42] **Health behaviour advice** [51, 57, 58, 62]

Generally, all SMS interventions included therapeutic education (A1); problem-solving therapy, goal setting and action planning (A12), including action plans (A3). In addition, various other strategies were used: evaluating non-traditional treatments, review (A4); feedback, monitoring adherence, support for self-monitoring (A5); medication management support (A6); SMS educational materials (A7); support for self-care (A10); practical support for self-management (A11); relaxation, deep breathing, positive thinking, motivational interviewing, behavioral activation, morale boosting, cognitive restructuring (A12); psychosocial interventions and social support (A13); brief interventions for misuse of alcohol or other substances and advice on healthy behaviors (A14).

### Integrated and non-integrated SMS interventions for CD and CMD (Question 1)

Five out of 10 interventions were considered integrated: TEAMcare [38–40], COMPASS [41–43], UPBEAT-UK [52–57], CAREplus [59, 60] and Langer [58]. However, all other studies had at least one category of integration or person-focused care: TEAMcare-PCN [65–67], Pathways [44–47], SCAMP [48–51], Trueblue [61, 62] and Step-Dep [63, 64] (see Table 5).

**Table 5:** Clinical integration of self-management support interventions by studies

**Study** Is the study integrated?	**Biopsychosocial perspective**	**Care based on needs, preferences and values**	**Co-creation of the care process**	**Shared responsibility and common agreement on clinical management**	**Person who coordinates his/her care when possible**
**SCAMP study** [48–51] Not integrated	No	No	No	Yes	Yes
**COMPASS study** [41–43] Integrated	Yes	Yes	Yes	Yes	Yes
**UPBEAT-UK study** [52–57] Integrated	Yes	Yes	Yes	Yes	Yes
**Pathways study** [44–47] Not integrated	No	No	No	Yes	Yes
**TEAMcare study** [38–40] Integrated	Yes	Yes	Yes	Yes	Yes
**TEAMcare-PCN** [65–67] Not integrated	No	Yes	Yes	Yes	Yes
**CAREplus study** [59, 60] Integrated	Yes	Yes	Yes	Yes	Yes
**Trueblue study** [61, 62] Not integrated	Yes	No	No	Yes	No
**Step-dep study** [63, 64] Not integrated	No	No	No	No	No
**Langer study** [58] Integrated	Yes	Yes	Yes	Yes	Yes

Yes: presence of clinical integration categories No: absence of clinical integration categories

### Characteristics of integrated and non-integrated SMS interventions (Question 2)

Several characteristics were noted for both integrated and non-integrated SMS interventions.

### Characteristics of integrated SMS interventions

Studies of integrated SMS interventions for CD and CMD presented biopsychosocial person-centered approaches (whole-person approach [60]; holistic [39]). These approaches were based on the person rather than on guidelines [55, 56], transcending physical and mental issues [58], where each CD and CMD was addressed during each SMS session. Education provided as part of SMS presented information about CD and CMD [38–40, 52, 58]. To achieve this, the UPBEAT-UK study adopted an individualized biopsychosocial plan, including a plan for each CD and CMD [41]. The SMS focused on several aspects of the individual, such as desires [39, 52], needs (personal, psychosocial, clinical) [42, 52, 58, 60], progress [60], priorities [59], concerns [59], preferences [42], challenges [42], strengths [42], readiness, health literacy and knowledge [42].

In terms of co-creation of the care process, integrated SMS interventions had several important characteristics. In general, the process of co-creating the SMS was patient-driven [39, 52, 58, 60]. The SMS was individualized to each person rather than based on a generic protocol [52] and the guidelines served as a recommendation for the nurse [39]. The nurse assisted the individual while retaining some authority over the SMS [39, 58]. In this sense, the SMS resulting from this co-creation was helpful [42], motivating, and adequately met the person’s needs [60]. The nurse-person relationship was an important feature of an integrated SMS. Establishing a partnership and collaborative peer-to-peer relationship was necessary [39, 42, 52]. The nurse was not solely focused on the disease and invested in the person by promoting their autonomy and valuing their ability to improve [58]. This relationship was made possible by developing the person’s trust [55], by taking the time to get to know the person [60], by engaging the person during the SMS and by making the person responsible for their health [58]. This relationship positioned the person as a key member of the team [38] and an expert in their health [42]. Certain attitudes displayed by the nurse, including being positive [39, 58], interested, encouraging, pleasant [57, 58], empathetic [60], authoritative, competent [58], and caring and connecting with the person [58, 60], seemed to foster co-creation of the care process. The person’s level of commitment was enhanced by organizing the SMS according to his or her motivation; preparing him or her; involving him or her in a meaningful way during the SMS (goal setting, action planning) and in the revision of the individualized plan [42].

Following the SMS sessions, nurses carried out an adapted follow-up, in agreement with the person [39, 42, 52, 60], with the possibility of adding sessions, depending on the person [39, 58]. Joint agreement was reached on several points: planning of the care process (SMS) [52]; common and mutual understanding of the problems and of the individualized plan [42, 60]; and on the person’s progress and follow-up [42]. Both the nurse and the person were responsible for the success in achieving and failure to achieve the goals of the plan [55]. The use of an individualized plan including biopsychosocial objectives that are set in conjunction with the person and other professionals, and written in the person’s own words, was an essential element of the process [38, 42, 52, 58, 60].

Finally, the practice of SM in the presence of the nurse [42, 58], the development of a sense of self-efficacy [52, 56], and the nurse’s encouragement of self-assessment [52] were all means of encouraging the person to coordinate his or her care independently. Contact between the nurse and the individual was planned to enhance autonomy and maximize SM practice when the patient’s condition permitted [42].

### Characteristics of non-integrated SMS interventions

Although a few studies of SMS interventions were not fully integrated for CD and CMD, some had features of clinical integration that were not named in the integrated SMS interventions. First, despite standardized approaches preventing full clinical integration of SMS, two interventions took a biopsychosocial approach [62, 65], and one offered SM materials (self-help course) tailored to comorbid clients [64]. Although a standardized intervention approach was implemented, it was possible for the individual to choose the initial treatment according to his or her needs [46, 51, 65]. In one study, satisfaction was one of the variables considered for treatment choice [47]. Although co-creation of the care process was not always present, adjustment of the standardized protocol by the nurse according to the person’s condition was a means of engaging the person during the SMS [61]. To ensure shared responsibility and agreement with the person, the treatment was negotiated with them [46], a shared individualized plan was developed with them and a copy was provided to keep them informed [50, 61, 66].

The non-integrated SMS intervention studies proposed highly standardized intervention approaches. Different approaches were used: disease-specific (treatment of only one CD/CMD); staged biopsychosocial (diseases treated separately in stages) [51, 64]; or indirect biopsychosocial (e.g., CMD addressed only if the CD is related to it) [44]. These approaches resulted in SMS sessions that did not address all diseases in one session and treated them separately, one at a time. In most studies of non-integrated interventions, SMS was not based on the individual’s needs, but rather on the protocol established by the study [45, 51, 61, 63], based on outcomes [45] or guideline-related risk factors [61]. In some cases, the person’s needs were not explicitly addressed [50, 61]. In fact, two studies reported that the intervention did not meet the needs of individuals [48, 63]. Co-creation of the care process was generally not present in these studies. In the organization of the SMS and its implementation, the person was not very involved in decisions and, in most cases, the established protocol was not adapted to the person [45, 50, 62, 64, 65]. Nurses offered few choices of SM strategies. Sometimes, it was not possible to deviate from the protocol and adopt different strategies or apply them at different times (e.g., at Step 2 instead of Step 3) [46, 63]. The number of SMS sessions was restricted, predetermined by the protocol and limited, with no room for adaptation [62, 63]. In these studies, the nurse-person relationship was poorly addressed [62, 63]. The nurse bore more responsibility for the SMS, including management of the individualized plan [61]. In several studies, it was unclear whether the individual independently coordinated their care [45, 61, 65].

## Discussion

This scoping review profiles studies of SMS interventions by primary care nurses for individuals with CD and CMD and describes important characteristics to consider when delivering SMS to ensure its clinical integration. Five out of 10 studies of SMS interventions fit all the categories of clinical integration as defined by Valentijn’s conceptual model (see Table 2) and were considered integrated. This synthesis identifies several features of integrated SMS, including the importance of the nurse-person relationship and a holistic approach to SMS, certain gaps in the theoretical underpinnings of SMS in the identified studies, and recommendations for future research and implementation projects.

Although not specific to the concepts of self-management [8, 68, 69] or SMS [31, 70–72], the relational aspect plays an important role in the clinical integration of SMS. Several qualitative studies address the nurse-person relationship as the focus of SMS. According to Harris et al. [73], a quality relationship based on mutual trust facilitates individualization of the SMS, communication, engagement, and would increase the person’s willingness to consider the nurse’s advice. In a qualitative study exploring how SMS should be applied in a multimorbidity clientele [74], the presence of a trusting relationship; an individualized SMS “by taking the patient’s agenda into account” (p. 6); relational continuity; and support “that went beyond information and disease management” (p. 6) are important elements. Another study reports similarities, noting that an SMS was perceived to be more effective in the presence of a needs-based (rather than disease-focused) relationship involving information exchange, negotiation and relational continuity [75]. On the other hand, this nurse-person relationship can be a source of conflict and vulnerability for each party [76, 77]. These sources of conflict are dependent in part on nurses and their definitions of autonomy and adequate SMS; and call for the use of a relational model for care, involving a sustainable relationship [76, 77]. Implementing a nurse-person relationship can also be challenging and will require a shift to less controlling, more collaborative clinical practices, and more room for the person [78, 79].

**Fig. 3:**
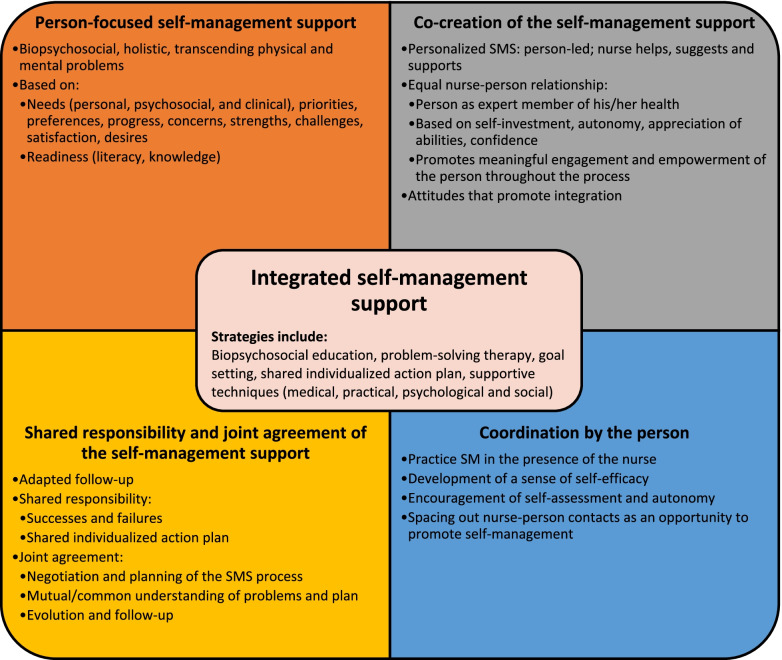
Integrated self-management support (adapted from Valentijn’s model [27])

Central to several models of person-centered and integrative approaches [27, 80–83], the biopsychosocial perspective is another defining feature of the integrated SMS, as it allows for the management of all health issues. However, it seems more difficult to apply a biopsychosocial SMS to each session and a few reasons may explain this: a lack of expertise in the field of psychosocial support and mental health; administrative priorities (e.g., data collection, funding) favoring physical CD; short encounters limiting holistic management; and predominantly biomedical clinical targets [23, 73, 74]. To improve the biopsychosocial approach and the clinical integration of SMS, the reviewed studies made several recommendations to enhance nurses’ training on the biopsychosocial approach [41, 44], patient engagement [41], behavioral change management and behavioral activation [41, 56], motivational interviewing [41, 44, 58], psychosocial support [41, 57], anxiety [57], and listening [60]. In addition, nurses should have skills and qualities that foster clinical integration, such as experience with people with CD and CMD, effective communication skills, motivation, confidence, competence, organization and adaptability [79]. Also raised in the literature [24], a better understanding of different therapeutic approaches and different combinations of SMS strategies would improve the effectiveness of SMS [44]. In light of this scoping review, several recommendations were made and to this we add that an integrated SMS intervention should have the elements described in Fig. 3, relying heavily on the different elements discussed in this scoping review.

In terms of SMS delivery, there was considerable variability in both the support strategies and the format used (frequency, duration, intensity). It was found that, at the very least, all SMS interventions included support, problem-solving, action planning and goal setting strategies, and educational interventions, which is consistent with the definition stated by the United States Institute of Medicine [13]. These are recognized as effective strategies [15, 20, 84]. However, sometimes, the description of SMS was not very detailed, and this deficiency can be attributed to the lack of theoretical references in the studies to clearly define the theoretical basis of SMS, as well as its components. Indeed, several studies present a collaborative care approach, based on the Chronic Care Model, which in turn also includes SMS [85], but none of them defined SMS using this model, nor did they refer to other authors or theoretical models to define its components. This lack of conceptual and operational clarity regarding SMS contributes to the heterogeneity of the concept, as well as to the wide variability in its application in intervention studies [35, 70]. In their international comparative analysis of different conceptual models framing CD SMS, O’Connell et al. [86] make a similar observation: Several elements that define SMS are similar between the reviewed documents, but few include references to theoretical foundations. In order to address this issue, different theoretical models have been proposed to better define and frame SMS, such as the PRISMS taxonomy of SMS [35, 72, 87–89]. Considering these theoretical gaps and the recent theoretical developments in SMS, it seems more important to emphasize that adopting a theoretical basis is essential to clearly define and frame the practice of SMS within a complex intervention and to allow for more focused and rigorous research on the subject [20, 90, 91].

In addition to these results, the scoping review led to two findings. First, the SMS was generally not very detailed in the studies, especially at the theoretical level and regarding its application, sometimes limiting the explanations to only a few lines. Editorial constraints, as well as a lower level of importance given to SMS during implementation, may have limited the explanations. However, two studies [39, 42] presented more detail about SMS because additional information was made available (e.g., training materials). These studies were helpful for this synthesis. Second, few of the studies were qualitative (*n* = 5 of 30). Specifically, there were no qualitative studies describing primary care nurses’ experience with SMS integration for individuals with CD and CMD.

### Practice implications

These results can guide primary care nurses towards better integration practices of SMS. Several recommendations were made for improving the nurse-person relationship, the biopsychosocial approach and clinical integration in general. At the clinical level, giving more importance to the development of the nurse-person relationship and to the various elements that foster it (relational continuity, commitment, accountability, self-investment, valuing, attitudes) will promote the clinical integration of SMS while having a beneficial effect for the person. Ensuring a biopsychosocial approach by personalizing the SMS to the person; adapting nurses’ training for clients with CD and CMD; increasing the duration of SMS meetings; and promoting an effective combination of SMS techniques are also elements to consider when implementing integrated SMS. Broadly, implementing these facilitators to clinical integration of SMS will require changes at the clinical and organizational levels. This will require the involvement of nurses, care recipients, and leaders.

### Strengths and limitations

This synthesis has some strengths. First, compared to the current literature [90, 92], to our knowledge, this scoping review is the first synthesis approaching SMS with an integrative view for CD and CMD. Second, the use of a recognized method enabled us to take a systematic approach and it gave us an overview of the literature on the topic [32]. The in-depth search strategy enabled us to find several additional relevant articles and the review and co-analysis process were conducted as a team.

However, this synthesis also has limitations. No protocol was established for this scoping review. Quality assessment of the included studies, which is not mandatory in scoping reviews [32], was not performed. As mentioned by other authors [93, 94], the heterogeneity of SMS may have influenced the number of articles identified, despite the use of several keywords and related terms. The sometimes limited description of the SMS may have influenced the identification or non-identification of certain elements of integration. Finally, this scoping review is a review of intervention studies that include SMS and the results may not fully reflect the natural clinical context. These results may provide guidance on the factors to consider in future research and during implementation in natural settings.

## Conclusion

This scoping review provided an initial overview of integrated and non-integrated SMS interventions provided by primary care nurses for people with CD and CMD, as well as identifying their main characteristics. The nurse-person relationship remains a central point in the clinical integration of SMS for this clientele. Many efforts need to be made to foster this relationship, as well as the active engagement of the person, requiring a change in SMS practice and a holistic approach. More effort is needed to better define integrated SMS theoretically and more qualitative research is needed to further explore nurses’ experience with clinical integration of SMS.

## Data Availability

The datasets used and/or analyzed during the current study are available from the corresponding author on reasonable request.

## Abbreviations

CD: Chronic diseases
CMD: Common mental disorders
SMS: Self-management support
